# Antibodies to a Full-Length VAR2CSA Immunogen Are Broadly Strain-Transcendent but Do Not Cross-Inhibit Different Placental-Type Parasite Isolates

**DOI:** 10.1371/journal.pone.0016622

**Published:** 2011-02-07

**Authors:** Marion Avril, Marianne J. Hathaway, Anand Srivastava, Sébastien Dechavanne, Mirja Hommel, James G. Beeson, Joseph D. Smith, Benoît Gamain

**Affiliations:** 1 Seattle Biomedical Research Institute, Seattle, Washington, United States of America; 2 Institut Pasteur, Unité de Biologie des Interactions Hôte-Parasite, Centre National de la Recherche Scientifique (CNRS), Unité de Recherche Associée (URA), 2581, Paris, France; 3 The Walter and Eliza Hall Institute of Medical Research, Parkville, Victoria, Australia; 4 Department of Global Health, University of Washington, Seattle, Washington, United States of America; 5 Institut National de la Transfusion Sanguine, Paris, France; 6 INSERM, UMRS 665, Paris, France; 7 Université Paris Diderot, Paris 7, Paris, France; The George Washington University Medical Center, United States of America

## Abstract

The high molecular weight, multidomain VAR2CSA protein mediating adhesion of *Plasmodium falciparum*-infected erythrocytes in the placenta is the leading candidate for a pregnancy malaria vaccine. However, it has been difficult so far to generate strong and consistent adhesion blocking antibody responses against most single-domain VAR2CSA immunogens. Recent advances in expression of the full-length recombinant protein showed it binds with much greater specificity and affinity to chondroitin sulphate A (CSA) than individual VAR2CSA domains. This raises the possibility that a specific CSA binding pocket(s) is formed in the full length antigen and could be an important target for vaccine development. In this study, we compared the immunogenicity of a full-length VAR2CSA recombinant protein containing all six Duffy binding-like (DBL) domains to that of a three-domain construct (DBL4-6) in mice and rabbits. Animals immunized with either immunogen acquired antibodies reacting with several VAR2CSA individual domains by ELISA, but antibody responses against the highly conserved DBL4 domain were weaker in animals immunized with full-length DBL1-6 recombinant protein compared to DBL4-6 recombinant protein. Both immunogens induced cross-reactive antibodies to several heterologous CSA-binding parasite lines expressing different VAR2CSA orthologues. However, antibodies that inhibited adhesion of parasites to CSA were only elicited in rabbits immunized with full-length immunogen and inhibition was restricted to the homologous CSA-binding parasite. These findings demonstrate that partial and full-length VAR2CSA immunogens induce cross-reactive antibodies, but inhibitory antibody responses to full-length immunogen were highly allele-specific and variable between animal species.

## Introduction

Pregnancy associated malaria (PAM) is a major cause of poor mother/child health and increases the risk for maternal anemia, prematurity, low birth weight and infant morbidity and mortality [1]. A key feature of PAM is the selective accumulation of *Plasmodium falciparum* infected erythrocytes (IEs) in the placenta mediated by adhesion to chondroitin sulfate A (CSA) [2]. Women become resistant to pregnancy malaria as they acquire antibodies to antigens expressed on the surface of IEs that inhibit placental adhesion and mediate opsonic phagocytosis [3]–[7].

VAR2CSA belongs to the *Plasmodium falciparum* erythrocyte membrane protein 1 (PfEMP1) family encoded by *var* genes [8]–[10] and is the leading PAM vaccine candidate [11]–[17]. The *var* gene family encodes cytoadhesion receptors that facilitate IE binding to microvascular endothelium through a variety of different host receptor interactions [18]. Adhesion of IEs to CSA or placental syncytiotrophoblast leads to specific transcriptional upregulation of *var2csa* [14]–[16] and native VAR2CSA binds to CSA [19]. Furthermore, VAR2CSA appears to be a major target of the antibody mediated immune response in pregnant women [15], [20], and parasites in which *var2csa* is genetically disrupted largely lose the ability to bind to CSA [12], [17] and placental syncytiotrophoblast [21], suggesting it is the primary *var* gene responsible for placental sequestration.

Key obstacles to the development of a pregnancy malaria vaccine are the limited understanding of the targets of inhibitory antibodies and the best way to induce these responses by vaccination. VAR2CSA is a large (∼350 kDa) and polymorphic protein with six different Duffy binding-like (DBL) domains and two other large interdomain regions [14], [22]. A significant component of the acquired antibody response to VAR2CSA appears to target polymorphic epitopes [20], many of which are shared between different VAR2CSA alleles due to extensive gene mosaicism [22]. This “patchwork” of polymorphic epitopes appears to contribute to cross-reactive antibody responses between different CSA-binding parasite lines [23]–[29], and may facilitate parasite escape from antibody-dependent protective mechanisms, as inhibitory epitopes appear to be at least partially strain-dependent [30]–[32]. Although CSA-binding properties have been mapped to several VAR2CSA domains using *in vitro* binding assays [13], [33], [34], recent published data [35], [36] casts doubt on the specificity of single DBL domains for CSA. Recently, the whole extracellular region of two different VAR2CSA variants were successfully produced as recombinant proteins and demonstrated to have significantly higher affinity and specificity for CSA than individual domains [37], [38], indicating that multiple domains may be involved in binding or come together to form a high affinity binding site(s). Furthermore, a low resolution structure of full-length VAR2CSA protein revealed a much more compact structure than predicted from x-ray crystallographic analysis of individual domains [38], consistent with three dimensional modeling that VAR2CSA surface polymorphism is biased with surprising amounts of invariant surface when individual domains are considered alone [22], [39]. Taken together, interactions between VAR2CSA domains are likely to occur and single domain recombinant proteins may display “off target” epitopes that are buried in the native protein.

While it has been possible to generate surface reactive antibodies with single-domain VAR2CSA recombinant proteins, it has been difficult to generate adhesion blocking antibody responses against individual VAR2CSA domains [23], [24], [29], [40]–[42]. To date, the most potent inhibitory antibodies response has been generated against the highly conserved DBL4 domain [43], but induction has not been consistently achieved against different VAR2CSA DBL4 alleles [42], [43]. Potential explanations are that single-domain immunogens may not possess the correct quaternary interactions of the native protein or reproduce the high affinity binding site(s). Thus, there is significant interest in characterizing larger, multidomain VAR2CSA immunogens that may better mimic the native protein structure. Whereas nearly all VAR2CSA vaccination studies have employed single domains, a recent study suggested that a full-length VAR2CSA recombinant protein was superior to the best single domain immunogen for inducing inhibitory antibodies [37]. However, the breadth of antibody inhibition against different CSA-binding parasite lines was not examined, and overall there has been limited characterization of multidomain VAR2CSA immunogens.

In this study, mice and rabbits were immunized with a partial length (DBL4-6) or a full-length VAR2CSA (DBL1-6) recombinant protein produced in the human embryonic kidney 293 cell line. Antibody cross-reactivity was assessed against individual VAR2CSA domains and on a diverse panel of CSA-binding parasite lines from different geographic regions for surface reactivity and binding inhibition activity.

## Methods

### Ethics statement

All animal work was conducted according to relevant national and international guidelines. Immunization studies were performed by custom vendors in France (Proteogenix) or the US (R&R Rabbitry). Animal experiments performed in France were approved and conducted in accordance with the Institut Pasteur or Proteogenix Biosafety Committee. Animals were housed under controlled laboratory conditions by qualified personnel who have obtained a license to do so from the French Agricultural Ministry (agreement B 75 15-08 dated May 22, 2008). All researchers performing animal experiments in this study were directly responsible for the experimental protocols and obtained individual licenses from the French Ministry of Agriculture. All animal experiments performed in the United States were approved by the Institute Animal and Care Use Committee at Seattle Biomedical Research Institute and at R&R Rabbitry. Immunizations were performed at R&R Rabbitry using the approved custom antibody protocol (Vendor's PHS assurance # A3982-01 and USDA registration 91-R-0038).

### Recombinant protein expression

The human embryonic kidney cell line (HEK293, Invitrogen) was used to produce 3D7 DBL4-6 and 3D7 DBL1-6 VAR2CSA (accession PFL0030c) proteins as soluble, secreted recombinant proteins [38]. IT4var18 DBL3 and VAR2CSA DBL recombinant proteins employed in the ELISA assay were produced in *Pichia pastoris* as previously described [23], [44].

### Animal immunization

Immunizations with partial (DBL4-6) and full-length (DBL1-6) VAR2CSA recombinant proteins were performed at Proteogenix, France, according to animal immunization guidelines. In brief, New Zealand White rabbits and Balb/c mice received recombinant protein in TiterMax Gold Adjuvant (Sigma) intradermally for first immunization and subcutaneously for three boosts. Rabbits received 50 µg of recombinant protein in first injection and 25 µg in subsequent injections while mice received 20 µg in first immunization and 10 µg in subsequent injections. A control rabbit plasma generated against the NTSDBL1 var O recombinant protein produced in *Baculovirus-*infected insect cells [45] was a gift from Odile Mercereau-Puijalon. This immune plasma was generated by immunizing rabbits with recombinant protein in Freund's adjuvant according to French animal immunization guidelines. Immunizations with IT4var18 DBL3 were performed at R&R Rabbitry (Washington, USA) according to animal immunization guidelines. Three New Zealand White rabbits received 50 µg of antigen in complete Freund's adjuvant for first immunization and were boosted with 50 µg of antigen in incomplete Freund's adjuvant. Plasma was heat-inactivated for 45 min at 57°C, preabsorbed on normal human red blood cell and stored at −20°C. IgG antibodies were purified from rabbit plasma using protein A columns (GE Healthcare, Sweden).

### ELISA assay

Rabbit and mouse plasma were tested against the homologous 3D7 DBL1 and 3D7 DBL5 recombinant proteins and heterologous 7G8 DBL1, 7G8 DBL3, 7G8 DBL4, 7G8 DBL5, and 7G8 DBL6 recombinant proteins using ELISA kit from Alpha Diagnostic International. 96-well ELISA plates (Nunc) were coated with 200 ng recombinant protein and incubated at 4°C overnight. After blocking, serial diluted plasma from 1/500 to 1/512,000 were added to antigen-coated wells in duplicate for 1 hr at room temperature followed by a 1/2000 dilution of HRP-conjugated goat anti-rabbit or anti-mouse IgG for 1 hr. Plates were washed and exposed to TMB substrate for 15 min, absorbance read at 450 nm and analyzed by SOFTmaxPRO version 5. Data were graphed with 4-pt fit curve and antibody titer calculated at 0.1 OD.

### Parasites lines

*Plasmodium falciparum* parasites were grown in O^+^ erythrocytes and 10% human serum. CSA-binding laboratory isolates, FCR3csa [46], 7G8csa [23], HB3csa allele A and HB3csa allele B [24], 3D7/NF54csa [31], Pf2004csa [24], [27] and Pf2006csa [24], [27] were maintained by periodic selection on CSA. Two CD36-binding negative control parasite lines, A4ultra and ItG-ICAM-1 were derived from the FCR3/ IT4 parasite genotype. Parasite genotyping was done with MSP1/MSP2 primers [47]. The primer set for MSP2 nested PCR is not conserved among Pf2004 and Pf2006 lines which explains the missing band (Figure S1). Transcription of *var2csa* was assessed by qRT-PCR using universal primers as described [23].

### Flow cytometry

Mature-stage IEs were grown in O^+^ blood and incubated with rabbit and mouse plasma that had been preabsorbed twice on uninfected O^+^ erythrocytes. For each assay, 10 million erythrocytes (5–8% trophozoites) were incubated with a 1/25 or 1/50 dilution of rabbit plasma or a 1/25 dilution of mouse plasma. Antibodies were detected with Alexafluor 488 conjugated goat anti-rabbit IgG (Molecular probes) or Alexafluor 488 conjugated goat anti-mouse IgG (Molecular probes). Samples were analyzed in an LSRII (Becton Dickinson) and analyzed using FLOWJO 8.1 software (Tree Star Inc). The adjusted mean fluorescence intensity (MFI)  =  (IE_i_-UE_i_) – (IE_p_–UE_p_) where IE_i_  =  MFI of infected erythrocytes following incubation in immune plasma; UE_i_  =  MFI of uninfected erythrocytes following incubation in immune plasma; IE_p_ =  MFI of infected erythrocytes following incubation in preimmune plasma; UE_p_  =  MFI of uninfected erythrocytes following incubation in preimmune plasma.

### Infected erythrocyte adhesion inhibition assay

IE binding was performed on bacterial petri dishes [23]. In brief, IEs were tested for binding to 10 µl spots of 0.05 mg/ml bovine CSA (Fluka Biochemika) for all parasite lines except for 3D7csa, which is a weaker binder [48] and required 0.1 mg/ml CSA. As a control, the ability of VAR2CSA plasma or purified IgGs to inhibit the binding of CD36-binding parasite lines to 0.05 mg/ml rCD36-Fc (R&D Systems) was employed. Prior to binding, IEs were enriched via pork gelatin flotation and parasitemias readjusted to 25% for all parasite lines. Binding assays were performed with 10 µl of 1×10^7^ IEs/ml per spot. Binding inhibition was calculated as percentage binding in presence of immune plasma relative to corresponding preimmune plasma control. Rabbit or mouse polyclonal plasma were tested at 1/5 and 1/10 final dilution or as plasma pool. IgG antibodies were purified from rabbit plasma using a protein A column (GE Healthcare, Sweden) and tested at a final concentration of 0.5 or 1.0 mg/ml. Purified IgG were dialyzed against PBS 1X and concentration determined by BCA assay (Pierce).

### Recombinant protein binding inhibition assay

Binding inhibition of full-length, HIS-tagged 3D7 VAR2CSA recombinant to CSA-proteoglycans was performed using ELISA plates coated with 100 µl/well of decorin (5 µg/ml) (Sigma) in PBS, overnight at 4°C. Plates were washed and blocked with PBS (0.05% Tween20 and 1% BSA) for 1 hr at 37°C. Recombinant protein 3D7 DBL1-6 (0.2 µg/ml) was pre-incubated with different concentrations of IgG protein A purified rabbit antibodies (0 to 100 nM) raised against full length 3D7 DBL1-6, 3D7 DBL4-6, or NTSDBL1 var O as a control for 45 mins at room temperature under constant shaking, and added to decorin coated plates for 1 hr at 37°C. Plates were washed 3X with PBS (0.05% Tween20). Binding of His-tagged DBL1-6 protein to decorin was detected by adding 100 µl of an HRP-labeled, anti-His monoclonal antibody (Qiagen) diluted 1∶2000 in blocking buffer for 1 hr at 37°C. TMB substrate (Biorad) was added to the wells before reading was measured at 655 nm.

## Results

### Domain specificity of antibodies generated to partial and full-length VAR2CSA recombinant proteins

**Figure 1:**
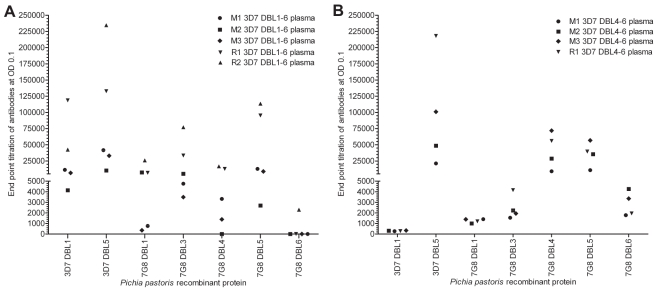
Immunization with DBL4-6 and DBL1-6 VAR2CSA recombinant proteins induces antibodies which target multiple domains in VAR2CSA. Mouse and rabbit plasma were screened by ELISA against different VAR2CSA recombinant proteins. (A) Rabbit and mouse plasma against the full-length DBL1-6 recombinant protein were screened against individual VAR2CSA domains, (B) rabbit and mouse plasma against DBL4-6 recombinant protein were screened against individual VAR2CSA domains. The end point titration of the corresponding preimmune plasma in ELISA ranged from 0 to 200 at OD 0.1. R1, rabbit #1; R2, rabbit #2; M1, mouse #1; M2, mouse 2; M3, mouse #3.

Mice and rabbits were immunized with either 3D7 DBL4-6 or 3D7 DBL1-6 recombinant proteins produced in HEK293 cells. The purified proteins migrated according to their expected molecular weight and were purified to higher than 95% purity [38]. In parallel, three rabbits received the *P. pastoris* protein IT4var18 DBL3 as a negative control [44]. To examine domain specificity, plasma were analyzed by ELISA against homologous and heterologous VAR2CSA-DBL domains produced in *P. pastoris* [44]. As expected, immune plasma to both immunogens reacted with 3D7 DBL5 recombinant protein, but only the anti-DBL1-6 plasma reacted with 3D7 DBL1 recombinant protein (Figure 1), indicating that multiple domains were targeted in the full-length immunogen and that antibody epitopes were not shared between the DBL4-6 domains and DBL1.

To examine cross-reactive epitopes in VAR2CSA, immune plasma were tested against five of the six DBL domains from the 7G8 VAR2CSA allele. It has not been possible to express the 7G8 DBL2 domain despite repeated attempts with different domain boundaries [44]. As shown in Figure 1, immunization with DBL1-6 recombinant protein induced cross-reactive antibodies to DBL1, DBL3, DBL4, and DBL5, but there was no or limited cross-recognition of 7G8 DBL6 in mice or rabbits immunized with either the DBL1-6 or DBL4-6 recombinant protein. The DBL6 domain is the least conserved of all VAR2CSA domains and maintains only ∼61% identity between alleles [22], which could contribute to the poor cross-reactivity. Another possible explanation for the poor recognition of VAR2CSA-DBL6 could come from the capacity of this domain to bind “non-immune” IgM [49], which could limit access of specific IgGs to their target epitopes. However, the VAR2CSA-DBL5 has also been reported to bind non-immune IgM [50], and this would not explain why there was good recognition of DBL5 in ELISA.

Of interest, both the partial and full-length recombinant proteins induced strong cross-reactive antibody responses to the 7G8-DBL5 domain. In contrast, the DBL4 domain, which is the best conserved var2CSA domains and also a target of inhibitory antibodies induced by immunization of rats [42], [43], was better recognized by all the animals immunized with the DBL4-6 recombinant protein compared to the DBL1-6 recombinant protein (Figure 1). To a lesser extent, this was also observed with the DBL6 domain (Figure 1). Thus, the immunogenicity of the DBL4-6 region differed between partial and full-length recombinant proteins. Taken together, both immunogens induced cross-reactive antibodies to one or more VAR2CSA domains, but there was weaker recognition of the DBL4 domain in animals immunized with the DBL1-6 recombinant protein.

### Immunization with full length or partial VAR2CSA recombinant protein induces a broad antibody response to diverse CSA-binding parasite lines

**Figure 2:**
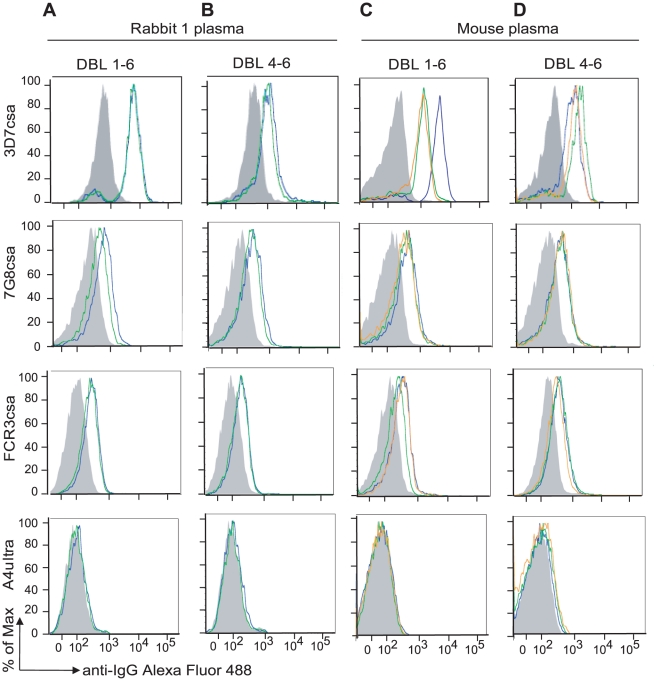
Immunization with DBL4-6 and DBL1-6 VAR2CSA recombinant proteins induces antibodies against the surface of IEs-CSA. Fluorescence of IEs labeled by rabbit plasma raised against 3D7 DBL1-6 (A) or 3D7 DBL4-6 (B) or mouse plasma raised against 3D7 DBL1-6 (C) or 3D7 DBL4-6 (D) is shown compared to the corresponding preimmune plasma (shaded histograms). In (A) and (B), blue histograms represent labeling with polyclonal plasma, green histograms show the corresponding purified IgG. In (C) and (D), individual mouse plasma were tested; blue (mouse 1), green (mouse 2), and orange (mouse 3).

To examine antibody reactivity with native protein, immune plasma were screened against the homologous 3D7csa parasite and a panel of six well characterized CSA-binding lines from diverse worldwide geographic areas, including South America (7G8), Central America (HB3), and West Africa (Pf2004 and Pf2006). Two CD36-binding control parasite lines that do not express VAR2CSA [24] were included as specificity controls. Prior to serological testing, all of the CSA-binding parasites lines in the panel were selected for CSA binding and confirmed by qRT-PCR for expression of the *var2csa* gene transcript as previously described [23]. All bound at a high level to CSA, except for the homologous 3D7/NF54 line which adheres to CSA at lower levels [24], [48]. *Plasmodium falciparum* parasites undergo chromosomal deletion or other changes under in vitro adaptation or long-term cultivation that can lead to a reduction in cytoadhesion capacity [51], [52] and the 3D7 parasite is known to be poorly cytoadhesive [48]. By comparing 3D7csa or the parental NF54csa parasite lines that were originally selected in different laboratories, we identified a 3D7csa parasite line [31] that was strongly reactive with anti-VAR2CSA antibodies (Figure 2) and bound at a moderate level to CSA. Like other 3D7csa or NF54csa parasite lines, the parasite quickly switched expression to a CD36 binding phenotype and it was necessary to maintain the CSA phenotype with frequent selections. All of the parasites in the panel were genotyped by PCR using MSP1/MSP2 primers during the course of the experiments to prevent cross-contamination (Figure S1) and, in addition, the authenticity of the 3D7csa parasite line was confirmed by sequencing PCR products from the highly polymorphic *Pfama1* and *Pfvar2csa* genes (data not shown).

Mouse and rabbit immune plasma against 3D7 DBL4-6 and 3D7 DBL1-6 recombinant proteins reacted by flow cytometry with homologous 3D7csa parasite line (Figure 2, Table 1), but did not react with the two CD36-binding control parasite lines (Figure 2, Table 1). In addition, immune plasma and purified IgG from mice and rabbits immunized with either the DBL4-6 or DBL1-6 recombinant proteins cross-reacted with multiple CSA-binding lines in the panel, although some parasite lines were recognized better than others (Figure 2, Table 1). In contrast, negative control rabbit antibodies (polyclonal and purified IgG) raised against IT4var18 DBL3 recombinant protein did not react with any of the parasites lines (Table 1). Therefore, native VAR2CSA protein at the IE surface contains cross-reactive epitopes targeted in both the DBL4-6 and DBL1-6 immunogens.

**Table 1:** Cross-reactivity of antibodies against full-length or partial VAR2CSA on lab-adapted lines.

			Fluorescence Intensity *								
			CSA-binding lines							Control lines	
Plasma		Dilution	FCR3	3D7	7G8	Pf2006	Pf2004	HB3A	HB3B	ITG-ICAM1	A4ultra
3D7 DBL1-6											
FB	M1	1/25	257	2338	292	136	417	142	141	3	2
FB	M2	1/25	133	795	204	82	200	434	478	4	2
FB	M3	1/25	257	718	187	121	277	145	132	1	1
FB	R1	1/50	392	5921	249	39	123	399	302	3	25
IgG	R1	0.11mg/ml	398	1818	148	43	178	603	256	3	20
FB	R2	1/50	429	472	165	149	221	397	nd	nd	6
IgG	R2	0.25mg/ml	527	415	89	88	321	477	nd	nd	6
3D7 DBL4-6											
FB	M1	1/25	139	767	200	102	245	169	157	0	6
FB	M2	1/25	169	1558	197	126	279	173	181	0	6
FB	M3	1/25	126	1044	251	99	244	190	228	0	0
FB	R1	1/50	285	1803	169	53	123	214	123	4	3
IgG	R1	0.15mg/ml	322	369	154	59	179	174	186	7	6
IT4var18 DBL3											
FB	R1	1/25	60	0	10	0	21	38	48	nd	0
FB	R2	1/25	0	20	0	0	0	4	0	nd	30
FB	R3	1/25	0	0	0	30	0	20	22	nd	5
IgG	R1	0.25mg/ml	18	0	0	0	0	0	0	nd	2
IgG	R2	0.25mg/ml	0	0	0	0	0	0	0	nd	0
IgG	R3	0.25mg/ml	0	0	0	0	0	0	0	nd	7

FB =  Final bleed M =  Mouse R =  Rabbit nd =  not done.

*Reactivity is expressed as the adjusted geometric mean of fluorescence intensity.

Values below the negative control anti-IT4var18 DBL3 plasma were considered negative.

### Immunization with full-length DBL1-6 VAR2CSA recombinant protein induces strain-specific adhesion blocking antibodies

**Figure 3:**
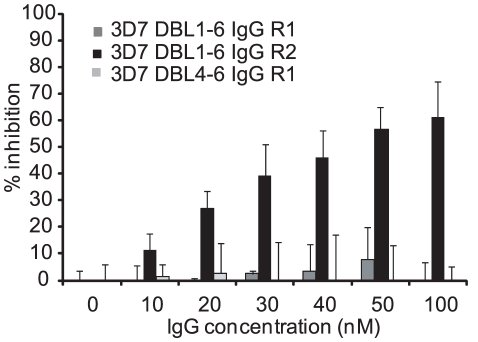
Antibodies against the full length protein inhibit 3D7 DBL1-6 VAR2CSA recombinant protein binding to CSA-containing proteoglycans. Purified antibodies were assessed for their capacity to inhibit protein binding to the CSA-containing proteoglycan decorin. Protein A purified rabbit IgG were preincubated at different concentrations with full-length recombinant VAR2CSA protein prior to addition to wells containing the CSA-containing proteoglycan decorin. Percent inhibition was calculated relative to the corresponding control protein A purified IgG raised against varO NTS-DBL1 domain. R1, rabbit #1; R2, rabbit #2.

**Figure 4:**
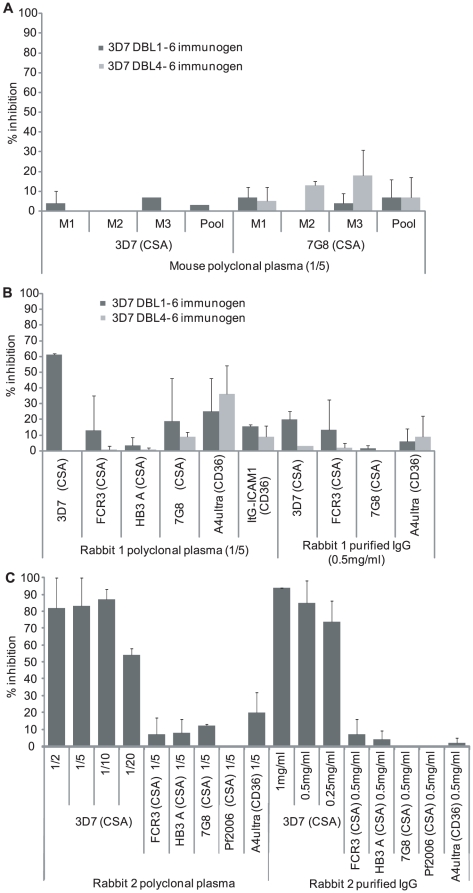
Immunization with DBL1-6 VAR2CSA recombinant protein induces strain-specific adhesion blocking antibodies. (A) Adhesion blocking properties of individual mouse plasma (1∶5 dilution) or pooled mouse plasma (1∶5 dilution) were tested against the 3D7csa and 7G8csa parasite lines. (B) Adhesion blocking properties of immune plasma (1∶5 dilution) or purified IgG (0.5 mg/ml) from rabbit #1 immunized with the DBL4-6 (grey bars) or DBL1-6 recombinant proteins (black bars) were tested against several different CSA-binding parasite lines and two CD36-binding negative control parasite lines. (C) Dose-dependency adhesion inhibition properties of rabbit #2 anti-3D7 DBL1-6 plasma and purified IgG tested against 3D7csa parasites. Rabbit #2 anti-3D7 DBL1-6 plasma (1∶5 dilution) and purified IgG (0.5 mg/ml) adhesion blocking properties were tested on four heterologous CSA-binding parasite lines and a CD36-binding negative control parasite line. For antibody adhesion inhibition, CSA-binding parasite lines were tested for adhesion to CSA and CD36 binding parasite lines were tested for adhesion to CD36. Percent inhibition was calculated relative to the corresponding preimmune rabbit plasma/purified IgG or a pool of three preimmune mouse plasma. Adhesion inhibition is the mean of 4 independent spots for all assays.

To evaluate binding inhibition activity, immune plasma and purified IgGs were tested in protein binding inhibition assays with the VAR2CSA DBL1-6 recombinant protein (Figure 3) and in IE adhesion inhibition assays (Figure 4). There was insufficient mouse plasma to evaluate in protein binding inhibition assays, so this was reserved for IE adhesion inhibition assays. The full-length VAR2CSA recombinant protein (DBL1-6) binds with nanomolar affinity to the placental CSA-proteoglycan [38]. In the protein binding inhibition assay, purified rabbit IgG against 3D7-DBL4-6 and a negative control varO DBL1 domain showed no binding inhibition and only purified IgG against full length 3D7-DBL1-6 inhibited binding of recombinant VAR2CSA DBL1-6 to the CSA-proteoglycan decorin (Figure 3). Binding was inhibited in a dose-dependent manner and rabbit 2 had much greater inhibition activity than rabbit 1 (Figure 3).

In IE adhesion assays, none of the immune plasma from the individual mice (M1, M2 or M3) or a pool of the three mouse plasma raised against either the partial or full-length VAR2CSA immunogens inhibited adhesion of 3D7csa-IEs to CSA (Figure 4A), although the M1 mouse plasma had much greater surface reactivity than M2 and M3 (Figure 2). Similar to the protein binding-inhibition assays, rabbit anti-DBL4-6 plasma or purified IgG had no inhibitory activity (Figure 4B), and only rabbits immunized with the DBL1-6 recombinant protein developed inhibitory antibodies (Figure 4B,C). Inhibition was dose-dependent and rabbit 2 had greater inhibition activity than rabbit 1 (Figure 4B,C), despite the fact that rabbit 2 had lower surface reactivity by flow cytometry (Table 1). In particular, rabbit 2 immune plasma could inhibit IE adhesion by greater than 80% at 1∶5 and 1∶10 dilutions (Figure 4C), while the inhibitory activity of rabbit 1 was 60% at 1∶5 dilution and was minimal at higher dilutions (Figure 4B, and data not shown). Similarly, purified IgG from rabbit 2 had significant inhibitory activity (greater than 70%) at concentrations as low as 0.25 mg/ml (Figure 4C), while purified IgG from rabbit 1 had little or no inhibitory activity at 0.5 mg/ml (Figure 4B).

To evaluate whether rabbit anti-DBL1-6 plasma or purified IgG could cross-inhibit binding of heterologous CSA-binding parasite lines expressing different VAR2CSA sequences, cross-inhibition experiments were performed against different CSA-binding lines. Despite reacting with these parasite lines by flow cytometry (Table 1), rabbit anti-DBL1-6 plasma or purified IgG did not significantly inhibit binding of heterologous CSA-binding lines to CSA compared to the binding of the negative control CD36 binding parasite line to the CD36 recombinant protein (Figure 4B,C). Therefore, immunization with the full-length VAR2CSA extracellular domain generated inhibitory antibodies against the homologous strain, but this inhibitory activity was highly strain-specific and restricted to rabbits.

## Discussion

Multiple lines of evidence suggest that VAR2CSA has an important role in placental binding and support its development for a pregnancy malaria vaccine, but protein size and polymorphism pose a challenge for vaccine development. Although women exposed to placental infections acquire antibodies that block adhesion of geographically diverse placental isolates [5], the specific targets of inhibitory antibodies remain unknown and inhibitory epitopes appear to be at least partially strain-dependent [30]–[32]. VAR2CSA is presumed to be an important target of inhibitory antibodies, but all of its extracellular domains are under diversifying selection [22] and it remains unclear which regions would make the best vaccine immunogen.

Because of its large size and multidomain architecture, initial vaccine development has concentrated on individual VAR2CSA DBL domains. While initial studies suggested that DBL2, DBL3, and DBL6 bind CSA [13], the specificity of these *in vitro* interactions has been questioned [36], and it is still unclear whether multiple individual domains independently engage CSA or if different domains come together to create one or more interaction site(s) in the native protein. This adds an extra layer of complexity in the choice of the immunogen(s) that would prevent placental sequestration. Whereas individual domains from a variety of heterologous expression platforms (*E. coli*, *Baculovirus-*infected insect cells, *P. pastoris* or HEK293) elicit antibodies that react with CSA-binding isolates [23], [24], [29], [40], [41], [53], only a few studies have reported inhibitory antibodies [41]–[43]. By DNA immunization, adhesion blocking responses were not observed against any of the VAR2CSA DBL domains [23]. In contrast, adhesion blocking responses have been generated to multiple different DBL domains produced in *Baculovirus-*infected insect cells, but the best and most consistent responses were generated in rats using the FCR3-DBL4 immunogen [42], [43]. These antibodies inhibited the binding of a range of clinical isolates from East and West Africa, although not all [54]. However, inhibitory antibodies were not elicited to three other VAR2CSA-DBL4 recombinant proteins produced in the same *Baculovirus* system [42], [43], suggesting that inhibitory epitopes may be poorly immunogenic or difficult to recapitulate with single domain immunogens.

Recent studies show that recombinant proteins based on the full-length VAR2CSA extracellular region containing all six DBL domains have ∼100,000-fold greater affinity than single DBL domains for CSA [37], [38]. Furthermore, the full-length recombinant protein had a much more compact structure than predicted from individual domains [38] and a DBL1-6 recombinant protein from the FCR3-CSA allele induced potent adhesion blocking antibody responses against the homologous CSA-binding parasite line that were almost 100-fold higher titer than the FCR3-DBL4 recombinant protein [37]. These findings raised the prospect that larger, multidomain or full-length proteins may be superior immunogens to single domains because they more closely mimic the native VAR2CSA structure.

In this study, we compared the immunogenicity of a 3D7 VAR2CSA DBL4-6 recombinant protein that binds with low affinity to CSA to a 3D7 VAR2CSA full-length DBL1-6 recombinant protein that binds with nanomolar affinity to CSA [38]. Both immunogens induced cross-reactive antibodies to different CSA-binding parasite lines, indicating they mimic native protein epitopes. By ELISA, several individual domains present in each immunogen were targeted by these antibodies. However, cross-reactive epitopes in the DBL4 domain were less efficiently targeted in all of the animals immunized with the full-length immunogen compared to the DBL4-6 recombinant protein. Furthermore, no inhibitory antibodies were obtained with the DBL4-6 recombinant protein. Considered with the fact that DBL4 has higher domain conservation [22] and that anti-DBL4 antibodies are frequently poorly reactive with the native protein [23], [40], [53] this suggests the DBL4 domain may be less accessible to antibodies in the native protein, which may contribute to difficulties in consistently generating adhesion blocking responses against this domain with single or multidomain immunogens.

Whereas recent work showed that potent adhesion blocking antibodies were generated in rats immunized with full-length FCR3-VAR2CSA recombinant protein [37], in this study, only rabbits and not mice developed inhibitory antibodies to a full-length 3D7-VAR2CSA protein. The reason why inhibitory antibodies are not consistently generated to full-length immunogens are unclear, but could relate to the recombinant protein preparation (FCR3 allele in *Baculovirus* insect cells versus 3D7 allele in mammalian cells), the animal species immunized, the adjuvant employed (Freund's complete/incomplete adjuvant versus TiterMax), or simply the size and complexity of full-length VAR2CSA immunogens. Our analysis indicates that multiple different DBL domains are targeted in multi-domain and full-length VAR2CSA immunogens. Although cross-reactive epitopes were recognized, antibodies did not cross-inhibit different CSA-binding parasite lines. These findings are reminiscent of an earlier immunization of rabbits with CSA-binding IEs [26], in which antibodies reacted with diverse CSA-binding lines [26] but only inhibited the homologous parasite isolate [31]. Thus, full length VAR2CSA recombinant protein is able to reproduce the immune response induced by CSA binding parasites, confirming the importance of VARCSA in the acquisition of cross-reactive antibodies. There are different potential explanations for why adhesion blocking responses were strain-specific, including that the interaction site(s) may differ between VAR2CSA alleles or there may be polymorphism surrounding a related binding site in different VAR2CSA alleles. Distinguishing between these possibilities will require a better understanding of how VAR2CSA binds CSA.

In conclusion, these findings demonstrate that immunization with full-length VAR2CSA extracellular region provides an experimental approach to generate adhesion blocking responses. This opens the way for future studies to dissect the mechanism of adhesion blocking. It further suggests that for larger multidomain immunogens it may be necessary to develop vaccine strategies that target the antibody response against critical adhesion blocking epitopes and that more than one allelic variant may be needed to cover polymorphism in the parasite population.

## Supporting Information

Figure S1 **Genotyping of parasite lines with MSP1 and MSP2 primers.** PCR products were run on agarose gels to distinguish different sized amplicons.
(EPS)
